# Photosynthetic bacteria-based whole-cell inorganic-biohybrid system for multimodal enhanced tumor radiotherapy

**DOI:** 10.1186/s12951-024-02654-7

**Published:** 2024-06-28

**Authors:** Shiyuan Hua, Jun Zhao, Lin Li, Chaoyi Liu, Lihui Zhou, Kun Li, Quan Huang, Min Zhou, Kai Wang

**Affiliations:** 1 Department of Respiratory and Critical Care Medicine, The Fourth Affiliated Hospital, Zhejiang University School of Medicine, Yiwu, 322000 China; 2 Department of Orthopedic Oncology, Spine Tumor Center, Changzheng Hospital, Naval Medical University, 415 Fengyang Road, Shanghai, 200003 China; 3 School of Basic Medicine, Tongji Medical College, Huazhong University of Science and Technology, Wuhan, 430030 Hubei China; 4 Health Science Center, Ease China Normal University, Shanghai, 200241 China; 5 University-University of Edinburgh Institute (ZJU-UoE Institute), Zhejiang University School of Medicine, Zhejiang University, Haining, 314400 China; 6 Institute of Translational Medicine, Zhejiang University, Hangzhou, 310009 China; 7 Zhejiang University-Ordos City Etuoke Banner Joint Research Center, Zhejiang University, Haining, 314400 China; 8 The National Key Laboratory of Biobased Transportation Fuel Technology, Zhejiang University, Hangzhou, 310027 China; 9 Department of Neurosurgey, The First Affiliated Hospital, Zhejiang University School of Medicine, Hangzhou, 320000, China

**Keywords:** Photosynthetic bacteria, Whole-cell inorganic-biohybrid system, Gold nanocluster, Type I photochemical mechanism, Radiotherapy

## Abstract

The whole-cell inorganic-biohybrid systems show special functions and wide potential in biomedical application owing to the exceptional interactions between microbes and inorganic materials. However, the hybrid systems are still in stage of proof of concept. Here, we report a whole-cell inorganic-biohybrid system composed of *Spirulina platensis* and gold nanoclusters (SP-Au), which can enhance the cancer radiotherapy through multiple pathways, including cascade photocatalysis. Such systems can first produce oxygen under light irradiation, then convert some of the oxygen to superoxide anion (•O_2_^−^), and further oxidize the glutathione (GSH) in tumor cells. With the combination of hypoxic regulation, •O_2_^−^ production, GSH oxidation, and the radiotherapy sensitization of gold nanoclusters, the final radiation is effectively enhanced, which show the best antitumor efficacy than other groups in both 4T1 and A549 tumor models. Moreover, in vivo distribution experiments show that the SP-Au can accumulate in the tumor and be rapidly metabolized through biodegradation, further indicating its application potential as a new multiway enhanced radiotherapy sensitizer.

**Supplementary Information:**

The online version contains supplementary material available at 10.1186/s12951-024-02654-7.

## Introduction

Microorganisms with photosynthetic capacity can effective harness solar power at low cost, and have gained popularity for applications in the fields of renewable energy and environmental protection [1–3]. The interaction of enzymes in microorganisms with inorganic materials showed higher photosynthetic efficiency than that of microorganisms alone. However, the catalytic function of enzymes often depends on the special environment and the synergistic effects of other proteins and organelles. Directly combining enzymes with inorganic materials will lead to a series of problems such as the poor stability. Using the enzyme mechanism in the whole cell can improve stability by preserving the innate replication and healing mechanisms [4]. Whole-cell inorganic biohybrid systems, including M. thermoacetica-CdS/Au NCs system [5, 6] and yeast-InP system [7], are coupled by complete cells and inorganic materials, which have low requirements for catalytic environment and can be stable production for a long time, further broadening the horizon of their potential usage [8–10]. In the field of medicine, microorganisms are mostly employed as drug carriers because of their targeted accumulation in lesions as well as their biodegradation capability [11–16]. It is unclear whether and how the whole-cell inorganic biohybrid system can be utilized for biomedical purposes.

Radiotherapy is a mainstay of tumoricidal modalities, especially for the local control of tumor or eradication of unresectable tumor nodules [17–20]. Its efficacy, however, is limited by collateral damage to the peritumoral healthy tissues and radio-resistance by tumor cells [21–23]. Radiosensitization is a common strategy in such context, so that lower radiation doses can be used to minimize radiotoxicity while not compromising the anti-tumor efficacy [24–26]. An important mechanism of radioresistance can be attributed to the hypoxic tumor microenvironment [27] and the abundance of intratumoral glutathione (GSH) [28], both of which diminish the amount of radiation-produced reactive oxygen species (ROS) radicals, attenuate DNA damage in tumor cells, and thereby protect tumor cells from radiotherapy [29]. Most of contemporary radiosensitizers, e.g. nitroimidazoles [30], exert their functions through the disruption of one single signaling pathway, often leading to the upregulation of compensatory signaling and subsequently adaptive resistance [31–33]. Simultaneous modulation of multiple radiation-related signaling pathways, therefore, is a challenge.

Microalgae is a naturally abundant microorganism with innate photosynthetic capacity, effectively produce oxygen upon irradiation with a photon source [34, 35]. It has been commercialized as nutrient supplements due to its satisfactory biocompatibility and pharmaceutical merits [36, 37]. We have previously shown that intratumoral hypoxia could be ameliorated using engineered photosynthetic microalgae systems, leading to improved anti-tumor efficacy of radiotherapy [38, 39]. Gold nanoclusters (Au NCs), on the other hand, have been extensively studied for biomedical applications in recent years due to their chemical versatility and biocompatibility [40–42]. Au NCs with a high Z-value (Z = 79) and a high attenuation co-efficient can substantially enhance the absorption of radiation energy in tumor tissues [43, 44]. Au NCs catalyze the conversion of hydrogen peroxide (H_2_O_2_) into hydroxyl radical (OH·), a type of reactive oxygen species that can induce DNA strand breaks, deplete intratumoral GSH, and synergize with radiotherapy [45–47]. Interestingly, Au NCs can also convert oxygen into superoxide anions (•O_2_^−^) through electron transfer, which have been reported even more toxic to DNA strands than type II ROS [48–51]. It remains unknown how a biohybrid system consisted of microalgae and Au NCs would affect tumor response to radiotherapy.

We hereby designed a whole-cell inorganic biohybrid system based on *Spirulina platensis*, a type of microalgae, and Au NCs. The resultant formulation, termed as SP-Au, sensitized radiotherapy through the following mechanisms (Sch. 1): (1) producing oxygen upon irradiation with red light and alleviating intratumoral hypoxia, (2) generating ROS radical (•O_2_^−^) and depleting GSH, and (3) absorbing radiation dose by Au NCs. Both intratumoral and intravenous injection of SP-Au enhanced radiotherapy in the 4T1 murine breast and A549 lung tumor model. SP-Au was rapidly metabolized and excreted through biodegradation after injection. Therefore, our results suggest that SP-Au is a promising radiosensitizer that simultaneously disrupt multiple signaling pathways.

**Sch. 1:**
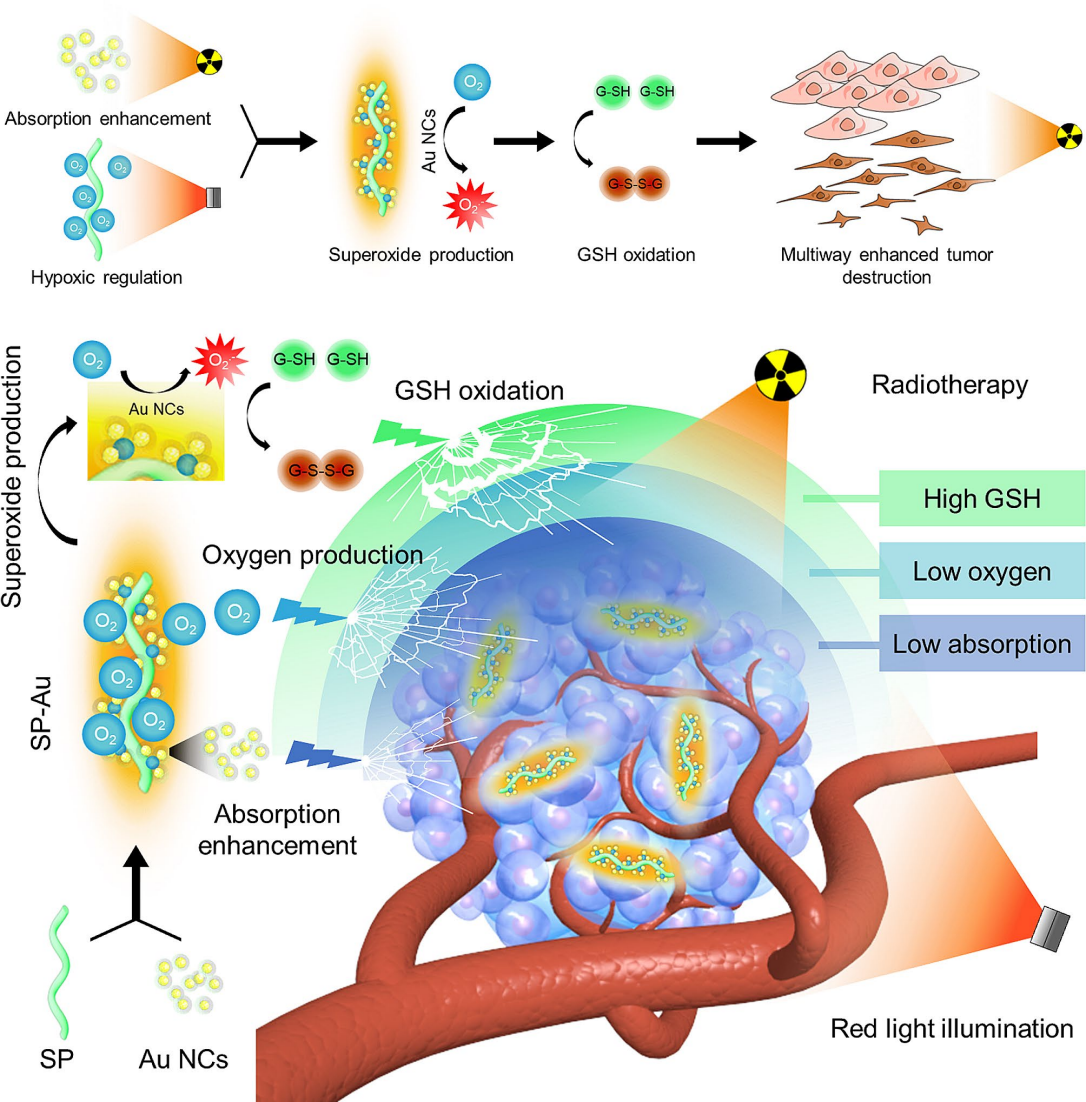
Schematic illustration of SP-Au mediated multiway enhanced radiotherapy. *Spirulina platensis* and gold nanoclusters based whole-cell inorganic-biohybrid systems could produce oxygen when irradiated with red-light to ameliorate tumor hypoxia, and then converted some of the oxygen to •O^2−^ by the catalysis of Au NCs, which could further decrease the GSH content in tumor cell. With the combination of hypoxic regulation, •O_2_^−^ production, GSH oxidation, and the radiotherapy sensitization of gold nanoclusters, the final radiotherapy was powerfully enhanced to kill tumor cells

## Experimental section

### Materials

All chemicals and reagents were used as received without any further purification. Carboxymethyl chitosan (CMCS, degree of substitution: ≥ 80%) and calcium chloride (CaCl_2_) were purchased from Macklin (Shanghai, China). Chloroauric acid hydrated (HAuCl_4·_H_2_O) and glutathione (GSH) were obtained from Sigma-Aldrich (St. Louis, USA). Dulbecco’s modified eagle’s medium (DMEM), phosphate-buffered saline (PBS), trypsin-EDTA, and fetal bovine serum (FBS) were purchased from Gibco-BRL (Burlington, Canada). De-ionized water (18.2 MΩ cm^− 1^) was prepared using a Milli-Q purification system (St. Louis, MO, USA) and used in all experiments.

### Synthesis of au NCs

HAuCl_4_ solution (4 mL, 50 mM) and GSH (6 mL, 50 mM) were added into the CMCS solution (80 mL, 1 mg/mL) with vigorous stirring at room temperature for 30 min, NaOH (2 M) was added to maintain the pH at 7.0. The solution was then heated at ∼ 70 °C for 9 h and gradually cooled to room temperature to yield a yellow solution. Au NCs were obtained and purified by ultrafiltration centrifugation.

### Synthesis of SP-Au

*S. platensis* sample was first collected by repeated centrifugation (4500 rpm, 10 min) and re-dispersion in 50 mL deionized water (DI water). It was then dispersed in 50 mL 1% CaCl_2_ solution and incubated for 30 min. After removing the unbound calcium ions by centrifugation, *S. platensis* was re-dispersed and stirred in 10 mL as-prepared Au NCs solution for 1 h to yield SP-Au [6, 52]. 

### Characterization

Optical and fluorescense images were captured by fluorescence microscope (Zeiss, Oberkochen, Germany). The morphology and EDS spectra of SP-Au were monitored with the transmission electron microscope (TEM, Hitachi HT7700, Japan) and scanning electron microscopy (SEM, HITACHI SU8010, Japan). Reactive oxygen species were analyzed using electron spin resonance (ESR, Bruker EMXplus-6/1, Germany). Optical absorption of SP-Au was measured on an ultraviolet, visible and near infra-red (UV-Vis-NIR) spectrophotometer (UV-2600, Shimadzu, Japan).

### Oxygen production of SP-Au

30 mL of SP, SP-Au (containing 1.5 mg SP) samples, or DI water were sealed in 50 mL centrifuge tubes in dark overnight to exhaust pre-dissolved oxygen. The tubes were then exposed to bright red light for 30 min, after which an oxygen sensing electrode (Unisense, Denmark) was used to measure the amount of produced oxygen. The standard curve of oxygen concentration was plotted using an oxygen saturated solution and an oxygen-depleted solution containing 0.1 mol /L ascorbic acid and 0.2 mol/L sodium hydroxide.

### Catalyticcapacity of SP-Au

The catalase-like activity of Au NCs and SP-Au was measured through the oxidization of 3,3′,5,5′-tetramethylbenzidine (TMB) by H_2_O_2_*via* UV-Vis-NIR spectrophotometer (UV-2600, Shimadzu, Japan). First, TMB (200 µL, 5 mM) and H_2_O_2_ (200 µL, 50 mM) were mixed with DI water, Au NCs (20 µg/mL), or SP-Au (equivalent to20 µg/mL Au NCs). The mixed solution was exposed to red light (LED light, 615 ∼ 650 nm, 4600 lx) for 15 min and scanned on a UV-Vis-NIR spectrophotometer. The production of superoxide anions was detected using 1,3-diphenylisobenzofuran (DPBF). DPBF solution (20 µL, 10 mM in ethanol) was added into 1980 µL of DI water, Au NCs (20 µg/mL) or SP-Au (equivalent to 20 µg/mL Au NCs) solution. The mixed solution was exposed to red light (615 ∼ 650 nm, 4600 lx) for 15 min and scanned on a UV-Vis-NIR spectrophotometer. The consumption of glutathione (GSH) was monitored using a glutathione detection assay kit (Solarbio, Beijing, China). In brief, DI water, Au NCs (20 µg/mL) or SP-Au (equivalent to 20 µg/mL Au NCs) was mixed with GSH (2 mM) and the final volume was adjusted to 2 mL using DI water. The mixed solution then was subjected to illumination using the red light for 15 min and centrifuged to remove catalyzers. The supernatant was then collected to measure the content of GSH.

### Cellular viability

3-(4,5-dimethylthiazol-2-yl)-2,5-diphenyltetrazolium bromide (MTT) assay was used to determine cellular viability. Briefly, HACAT keratinocytes, HEK293 human embryonic kidney cells, 4T1 murine breast cancer cells, and A549 murine lung cancer cells were seeded in 96-well plates overnight at 8 × 10^3^ cells per well. A 100 µL suspension of SP-Au in complete growth medium at different concentrations (0, 6.25, 12.5, 25, 50,100, or 200 µg/mL) was added to each well followed by a 24-h incubation. After aspiring the treatment solution and washing with phosphate buffered saline (PBS), the MTT working solution was added and incubated for 4 h. The supernatant was aspirated and dimethyl sulfoxide (DMSO) was added to dissolve the insoluble formazan product. The cellular viability was measured using the absorbance at 490 nm on a multifunctional plate reader (MD M5, Molecular Devince, San Jose, USA).

### In vitro catalytic performance of SP-Au

4T1 cells were seeded in 96-well plates at 8 × 10^3^ cells per well overnight in a hypoxic incubator (1% O_2_). Blank DMEM medium, Au NCs (20 µg/mL), or SP-Au (equivalent to 20 µg/mL Au NCs) was added and the plates were illuminated under 4600 lx red light (615 ∼ 650 nm) for 15 min. The cells were then stained with superoxide anion selective dihydroethidium (DHE), and visualized under a fluorescence microscope (Zeiss, Oberkochen, Germany).

### In vitro evaluation of SP-Au based radiosensitization

4T1 cells were seeded in6-well plates at 2 × 10^5^ cells per well and incubated overnight at 37 °C in in a hypoxic incubator (1% O_2_). DMEM medium, Au NCs (20 µg/mL), or SP-Au (equivalent to 20 µg/mL Au NCs) was then added with or without GSH (2 mM), and illuminated with 4600 lx red light (615 ∼ 650 nm) for 15 min. The plates were exposure to X-ray at doses of 0, 3, 6, and 9 Gy, respectively. The cells were culture for 7 more days for the formation of colonies (≥ 50 cells), which were subsequently stained with Giemsa and counted. The produced ROS was stained right after irradiation at a dose of 6 Gy using a DCFH-DA assay kit (YEASEN, Shanghai, China) The staining of Live/dead cells, were conducted using a Calcein-AM/PI double stain kit (YEASEN, Shanghai, China).

### In vivo biodistribution of SP-Au

Animal studies were approved by the Institutional Animal Care and Use Committee of Zhejiang University. Balb/c mice bearing 4T1 tumors were injected intravenously (*i.v.*) with SP-Au (150 µL, 200 µg mL^− 1^), and then monitored on an IVIS Lumina LT Series III scanner (Perkin Elmer, Massachusetts, USA) at 0.5, 1.5, 2.5, 4, 7, and 24 h post-injection. The tumors and major organs (heart, liver, spleen, lung, and kidney) were then collected and imaged ex vivo at 2.5, 7 and 24 h post-injection.

### In vivo biodegradability of SP-Au

SP-Au sample (100 µg/mL) was suspended in DMEM solution, added into 24-well plates pre-seeded with 1 × 10^5^ 4T1 cells per well, and then incubated in a 5% CO_2_ atmosphere at 37 °C. After 6 h, the *SP-Au* samples were imaged under a fluorescence microscope (Zeiss, Oberkochen, Germany). To investigate the renal clearance of SP-Au, mice were intravenously injected with SP-Au (150 µL, 200 µg/mL). At 0, 3, 6, 12, 24, 48, and 72 h post injection, urine samples were collected and characterized on an RF-6000 fluorescence spectrophotometer (Shimadzu, Kyoto, Japan).

### In vivo catalytic performance of SP-Au

4T1 tumor-bearing mice were injected intravenously with 150 µL PBS, Au NCs (40 µg/mL); SP (200 µg/mL), or SP-Au (equivalent to 40 µg/mL Au NCs) [38, 52]. At 2.5 h post-injection, the mice were illuminated with red light (615 ∼ 650 nm, 4600 lx) for 15 min. Subsequently, mice in Light + RT, Au + Light + RT, SP + Light + RT and SP-Au + Light + RT groups were further treated with 6 Gy X-ray irradiation. After 30 min, the 4T1 tumors were collected for the preparation of frozen sections and staining with DHE or DCFH-DA.

### Radiosensitization of intratumorally/intravenously injected SP-Au

Two tumor models, 4T1 and A549, were used to validate the intratumoral and intravenous administration of SP-Au, respectively. BALB/c mice were used for the 4T1 tumor model and BALB/c nude mice were used for the A549 tumor model. Mice bearing 4T1 or A549 tumors were randomly allocated into eight groups once the tumor volume reached 100 mm^3^: Control, Au, SP, SP-Au, RT, Au + RT, SP + RT and SP-Au + RT (*n = 5* per group). Mice in the control and RT group were injected intratumorally/intravenously with 50/150 µL saline. Mice in the Au, SP, SP-Au, Au + RT, SP + RT and SP-Au + RT groups were injected intratumorally with 50/150 µL of respective catalysts: Au NCs (40 µg/mL), SP (200 µg/mL); or SP-Au (40 µg/mL) [38, 39, 52]. At 2.5 h post-injection, the mice were illuminated with red light (615 ∼ 650 nm, 4600 lx, 15 min), and those in RT, Au + RT, SP + RT, and SP-Au + RT groups were irradiated with 6 Gy X-ray. Tumor size was measured using a digital caliper and calculated as volume (mm^3^) = length × width^2^ × 0.5. All the mice were sacrificed on day 18 (4T1 tumors) or day 10 (A549 tumors) after enrollment. Tumors and major organs (heart, liver, spleen, lung, and kidney) were excised and fixed in 4% paraformaldehyde. Hematoxylin and eosin (H&E) sections were scanned on a virtual slide microscopy (Olympus VS120, Olympus Life Sciences, Waltham, MA, USA). Unstained sections were stained for CD31, Ki-67, or HIF-1*α*.

### In vivo toxicity of SP-Au

Mice (*n = 3*) were intravenously injected with 150 µL of PBS or SP-Au (500 µg/mL), and sacrificed 24 h later. Blood samples were obtained for routine blood chemistry and biochemical analysis.

### Statistical analysis

All data were presented as the mean ± standard deviation or mean. Statistical significance was determined using Student’s t-test. *P* values less than 0.05 were considered statistically significant and indicated in Fig.s and/or legends as ***P < 0.001; **P < 0.01; *P < 0.05.

## Results and discussion

### Bioengineering and characterization of SP-Au

**Fig. 1:**
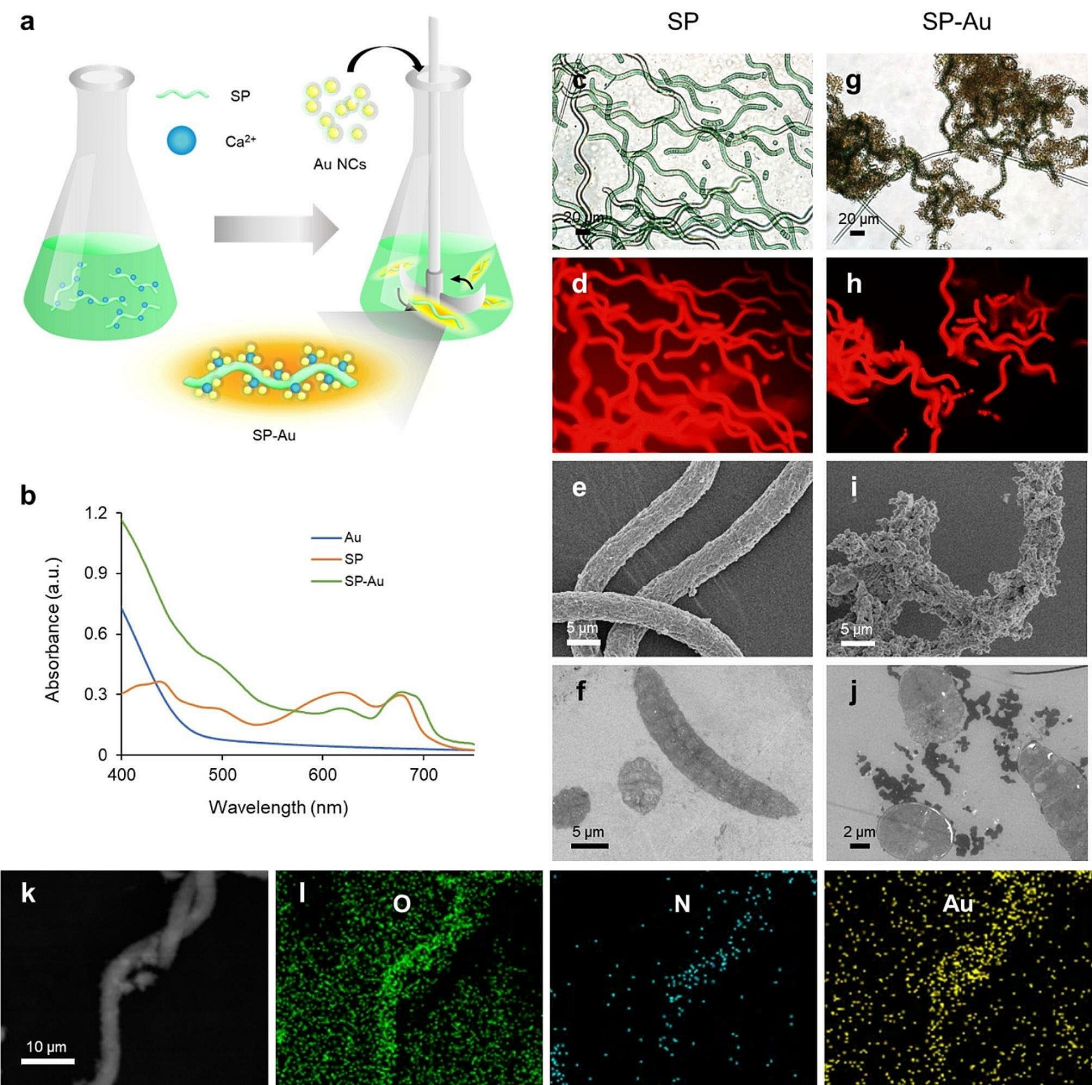
Characterization of the SP-Au biohybrid System. (**a**) Schematic illustration for the synthesis of SP-Au. (**b**) UV–Vis-NIR spectra of Au, SP, and SP-Au. Representative (**c**) Bright-field, (**d**) fluorescence, (**e**) SEM and (**f**) TEM images of SP. Corresponding (**g**) Bright-field, (**h**) fluorescence, (**i**) SEM and (**j**) TEM images of SP-Au. (**k**) Dark field and (**l**) elemental mapping (O, N, Au) images of SP-Au

*Spirulina platensis* (*S. platensis*, abbreviated so forth as SP) is a type of natural microalgae and is composed of multicellular helical structures. It could effectively produce oxygen under light illumination, and spontaneously emit red fluorescence signals due to its constituent chlorophyll. Therefore, SP has the potential to be used as imaging tracers in vivo. To prepared the biosystem doped with ultrasmall gold nanoclusters (Au NCs, abbreviated as Au so forth), SP was first incubated with a solution of calcium chloride (CaCl_2_), and then mixed with carboxymethyl chitosan-coated Au NCs [52]. (Fig. 1a). The Au NCs were then adsorbed onto the surface of SP through the ionic crosslinking of Ca^2+^ ions. As shown in Fig. 1b, the UV-Vis spectrum of SP-Au exhibited significantly higher absorption between 400 and 550 nm than that of SP, which was in the similar pattern to that of Au NCs, indicating a successful loading of Au NCs on SP. The substantial coating layer on the SP helical structures was easily recognizable when observing SP-Au under a bright-field or fluorescent microscope (Fig. S1 & Fig. 1c, g,d, h). SEM also revealed granular structures on the surface of SP-Au, in contrast to the smooth surface of SP (Fig. S2 & Fig. 1e, i). TEM, on the other hand, showed that SP-Au exhibited substantially higher contrast than SP alone, which can be attributed to the presence of Au NCs (Fig. S3 & Fig. 1f, j). The successful coating of Au NCs was further verified using energy dispersive spectroscopy (EDS), where the distribution of Au element colocalized with those of oxygen and nitrogen in SP (Fig. 1k, l). In summary, we have successfully prepared SP-Au.

### Oxygen production and catalytic performance of SP-Au

**Fig. 2:**
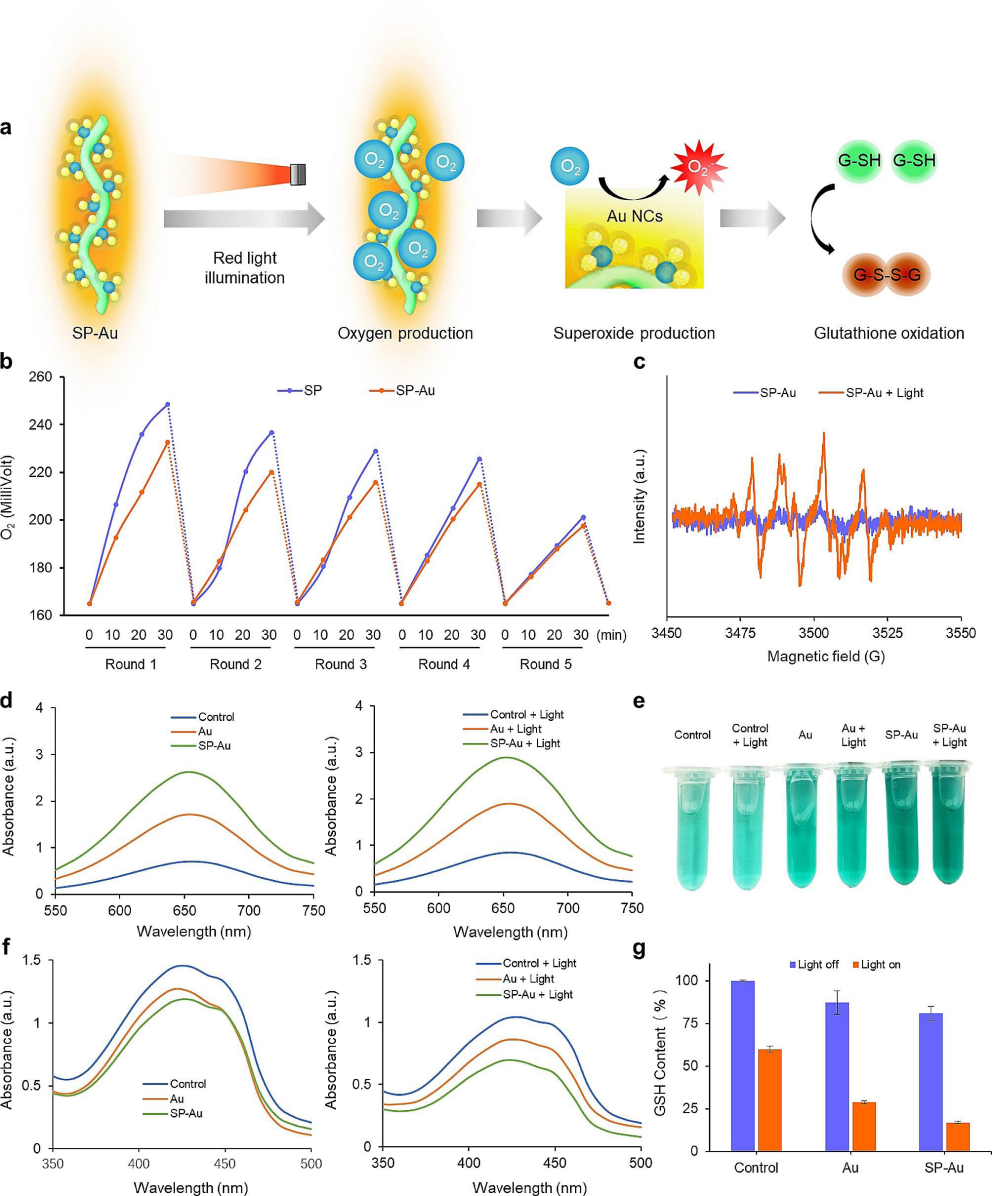
SP-Au based oxygen production and catalytic activity study. (**a**) Schematic illustration for the oxygen and superoxide anion production of SP-Au and glutathione oxidation. (**b**) Curves of oxygen concentrations in SP and SP-Au solutions under the light irradiation in 30 min. The experiment was repeated after the solutions being placed in dark environment overnight. (**c**) ESR spectra of •O_2_^−^, generated by SP-Au before and after red light treatment (615 ∼ 650 nm, 4600 lx, 15 min). (**d**) Absorption spectra and (**e**) Optical images of the oxidized TMB produced by different catalyzers before and after red light illumination (615 ∼ 650 nm, 4600 lx, 15 min). (**f**) Absorption spectra of the DPBF before and after red light treatment (615 ∼ 650 nm, 4600 lx, 15 min). (**g**) GSH consumption with different catalyzers before and after red light illumination (615 ∼ 650 nm, 4600 lx, 15 min). All data were presented as the mean ± standard deviation

We next investigated the oxygen production and catalytic activity of SP-Au. Upon illumination with red light, oxygen was generated by SP through photosynthesis. The oxygen was then converted to •O_2_^−^ under the catalysis of Au NCs, which can further oxidize GSH in the solution (Fig. 2a). Indeed, the oxygen concentration in both SP and SP-Au solutions increased up to 1.5 times that of baseline within 30 min of illumination (Fig. 2b). The oxygen production was basically remained after at least 5 cycles of light illumination, indicating the relatively photostability of SP-Au. In addition, the oxygen concentration in the SP-Au solution was slightly lower than that in the SP solution, which can be attributed to the catalytic conversion of oxygen toward •O_2_^−^ by Au NCs. The catalytic activity of SP-Au was further verified using 3,3′,5,5′-tetramethylbenzidine (TMB, detecting catalase-like activity) and 1,3-diphenylisobenzofuran (DPBF, detecting superoxide anion) probes [53]. As shown in Fig. 2d, e, the absorption of oxidized TMB at 650 nm increased when the solutions of SP-Au and Au NCs were illuminated with red light for 15 min. Accordingly, the absorption of DPBF decreased (Fig. 2f). Interestingly, SP-Au was more capable of producing ROS than Au, probably because extra oxygen was produced by SP-Au under illumination. Electron spin resonance (ESR) analysis revealed the ROS produced by SP-Au under red light illumination was mostly •O_2_^−^, while •OH or ^1^O_2_ was barely present (Fig. 2c & Fig. S4). The •O_2_^−^ produced by SP-Au or Au NCs oxidized GSH in the solution (Fig. 2g). In summary, we have shown that SP-Au generated oxygen under red light illumination, which was converted to •O_2_^−^ by the constituent Au NCs. The •O_2_^−^ subsequently oxidized GSH while exerting the photodynamic therapeutic effects.

### In vitro radiosensitization

**Fig. 3:**
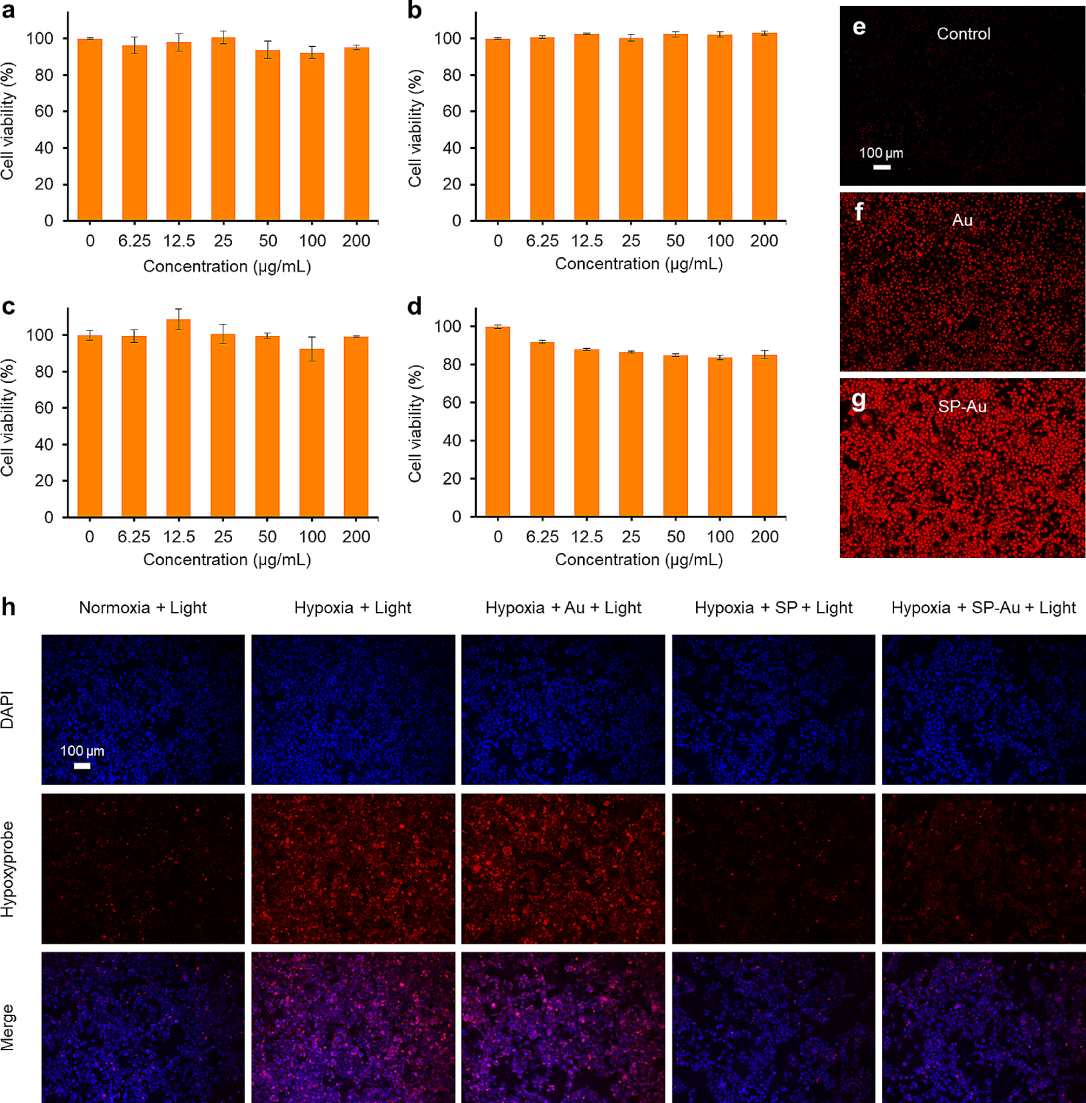
In vitro cytotoxicity, hypoxia regulation and catalytic performance of SP-Au. (**a-d**) Cell viabilities of HACAT keratinocytes, kidney 293 cells, 4T1 and A549 cancer cells after incubation with series concentrations of SP-Au for 24 h, respectively. (**e-g**) Representative fluorescence images of DHE-stained 4T1 cancer cells (red, •O_2_^−^) with different catalyzers after red light treatment (615 ∼ 650 nm, 4600 lx, 15 min). (**h**) Representative fluorescence images of 4T1 cancer cells stained with DAPI (blue, nuclei) and hypoxyprobe (red, hypoxic cells) after different treatments. All data were presented as the mean ± standard deviation

The cytotoxicity of SP-Au was first evaluated. No obvious toxicity was observed after by 24 h incubation of SP-Au with HACAT keratinocytes (Fig. 3a), HEK 293 cells (Fig. 3b), 4T1 murine breast cancer cells (Fig. 3c), and A549 lung cancer cells (Fig. 3d) at different concentrations. The DHE fluorescent probe, which detects intracellular •O_2_^−^, was added to cell culture containing Au NCs or SP-Au. Only the SP-Au-containing cells exhibited a strong red fluorescence (Fig. 3e-g). The capacity of SP-Au to alleviate hypoxia was assessed under a hypoxic condition (1% O_2_) and measured using the [Ru(dpp)_3_]Cl_2_ hypoxic probe [35, 36]. Cells treated with SP or SP-Au exhibited significantly weaker fluorescence than the other groups, which was almost similar to that of the normoxia control (Fig. 3h). Therefore, we have established that SP-Au could efficiently produce oxygen to alleviate hypoxia and converted to •O_2_^−^ under red light illumination.

**Fig. 4:**
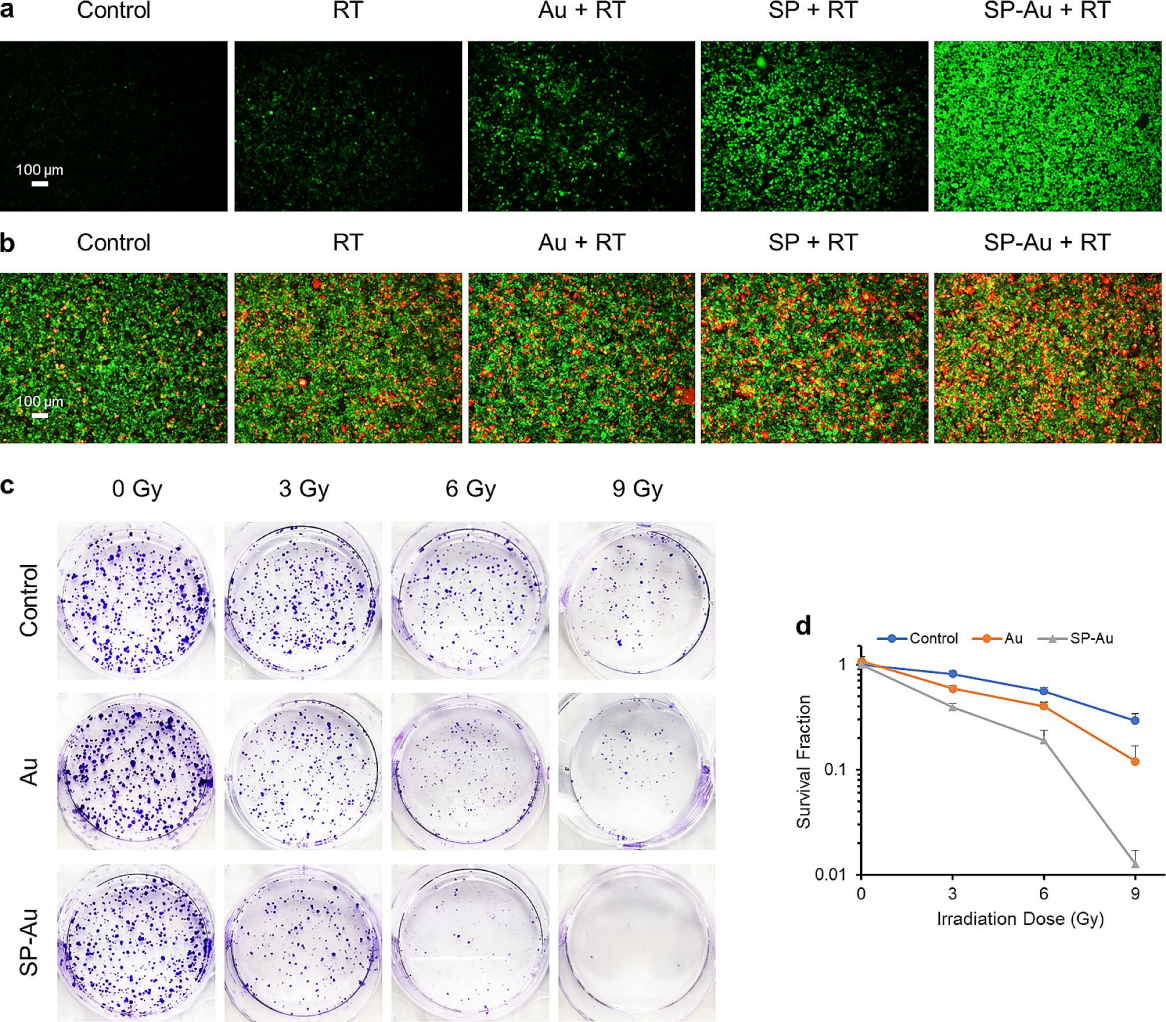
In vitro evaluation of SP-Au based multiway enhanced radiotherapy. (**a**) Representative fluorescence images of DCFH-DA-stained 4T1 cancer cells (green, ROS) after red light treatment (615 ∼ 650 nm, 4600 lx, 15 min) and X-ray radiation (6 Gy). (**b**) Representative fluorescence images of 4T1 cancer cells co-stained with calcein-AM (green, living cells) and PI (red, dead cells) after different treatment. (**c**) The colony assay of 4T1 cancer cells incubated with different catalyzers under X-ray radiation (0, 3, 6, and 9 Gy). (**d**) Corresponding survival fractions of 4T1 cancer cells incubated with different catalyzers under X-ray radiation (0, 3, 6, and 9 Gy). All data were presented as the mean ± standard deviation

The radiosensitization of SP-Au was first investigated in vitro. Cells treated with Au NCs or SP along with red light illumination exhibited higher ROS concentrations than those treated with RT alone. Interestingly, cells treated with SP-Au + illumination and RT had the highest ROS content, probably because of the additional •O_2_^−^ produced by SP-Au (Fig. 4a). The calcein AM/PI double staining was then used to differentiate live (green) and dead (red) cells after different treatments (Fig. 4b). 4T1 cells co-incubated with SP-Au and then subjected to red light illumination and a 6-Gy radiation exhibited significantly more red cells than the other groups. Colony formation assay was further performed under hypoxic condition (Fig. 4c, d). Adding Au NCs and red-light illumination only marginally sensitized the cells to radiation, indicating the radio-resistance by hypoxia and GSH. In contrast, adding SP-Au and red-light illumination significantly reduced cell survival under radiation, and the survival fraction was only 0.19 that of control at the 6-Gy dose. In summary, SP-Au enhanced cellular response to radiotherapy through the production of oxygen and •O_2_^−^ as well as the intrinsic radiosensitizing effect of Au NCs.

### Tumor accumulation and biodegradation of SP-Au

**Fig. 5:**
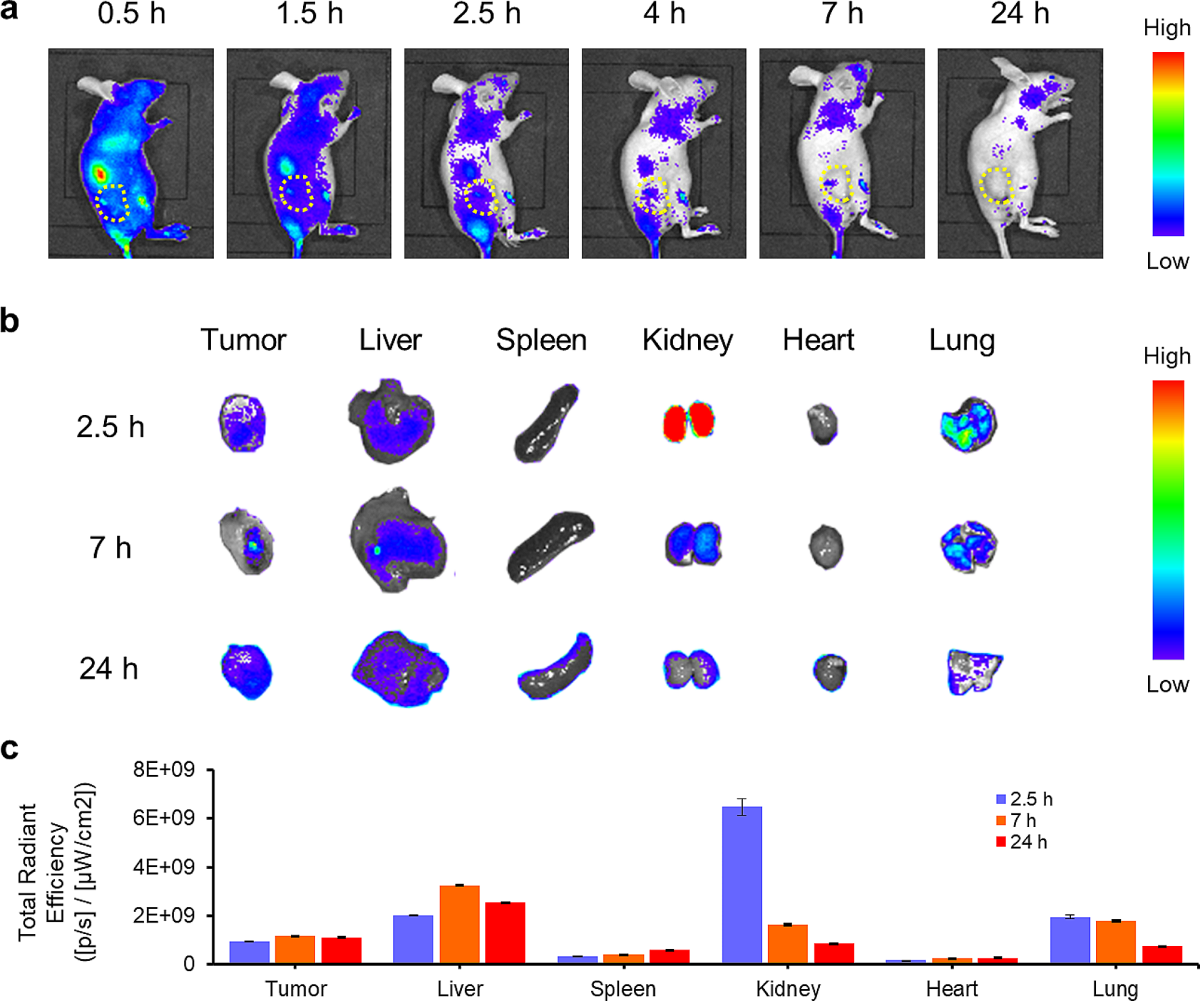
In vivo biodistribution and tumor accumulation of SP-Au. (**a**) In vivo fluorescence imaging of 4T1 tumor-bearing mice at different time points (0.5, 1.5, 2.5, 4, 7 and 24 h) after *i.v.* injection of SP-Au (tumor, yellow circle). (**b**) Ex vivo fluorescence imaging of tumors and major organs collected from 4T1 tumor-bearing mice at different time points (2.5, 7 and 24 h) after injection of SP-Au. (**c**) Quantitative analysis of fluorescence signals in tumors and major organs at different time points (2.5, 7, and 24 h). All data were presented as the mean ± standard deviation

The biodistribution of SP-Au was first examined by measuring its red fluorescence signals after intravenous injection into mice bearing 4T1 tumors. The whole-body imaging showed the strongest fluorescence at 30 min post-injection, which then gradually decreased over time (Fig. 5a). Notably, fluorescence signal could be identified at the tumor region starting from 2.5 h post-injection, and persisted until 7 h post-injection. Ex vivo scanning of excised organs also revealed an effective tumor-uptake of SP-Au from 2.5 to 24 h post-injection. The fluorescence signals in lung and kidneys peaked at 2.5 h post-injection, which when decreased over time, indicating a possible metabolism and subsequent excretion of SP-Au (Fig. 5b, c). The uptake of SP-Au in liver was the highest at 7 h post-injection, which then slightly decreased at 24 h.

**Fig. 6:**
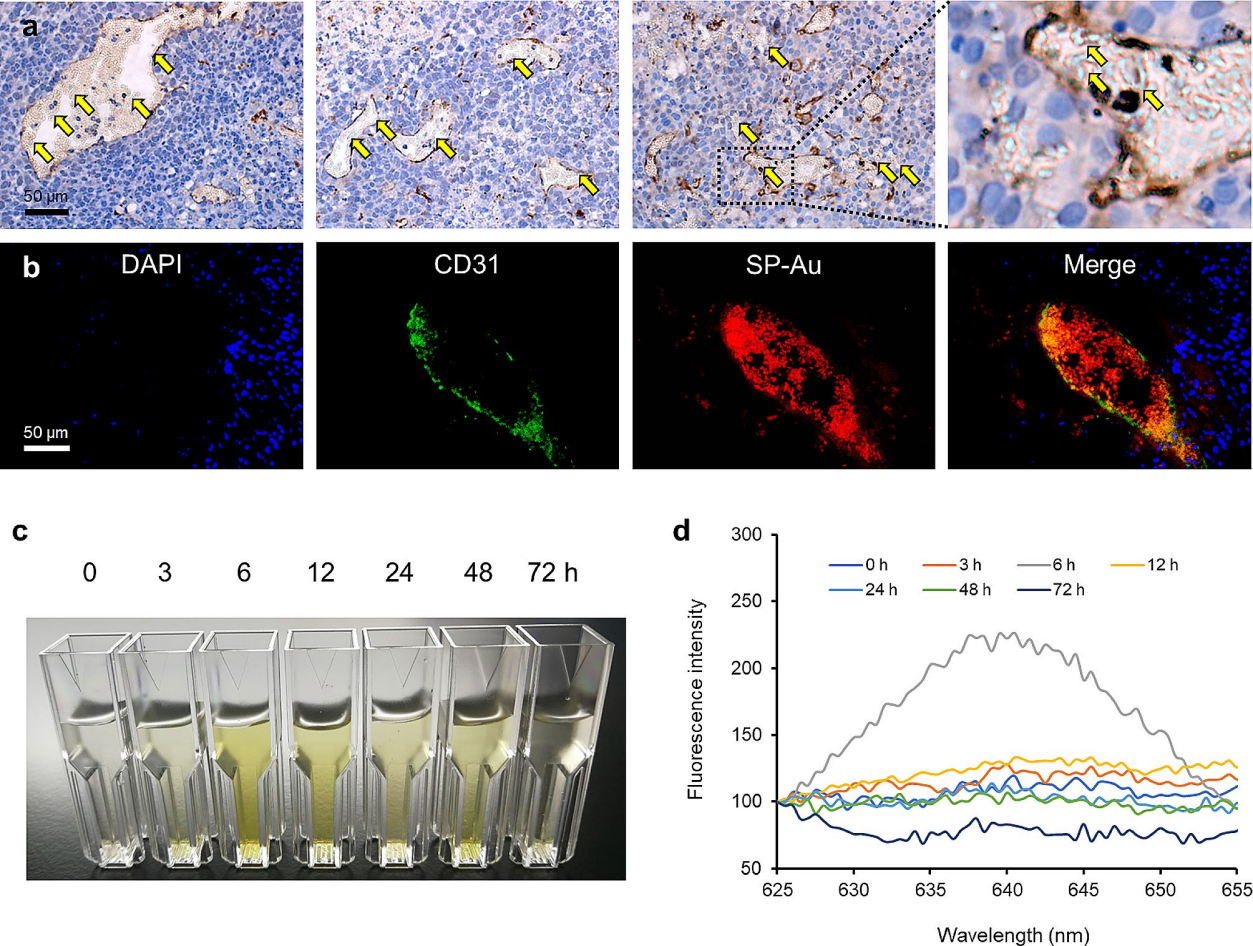
In vivo tumor accumulation and biodegradability of SP-Au. (**a**) Representative CD31 staining of tumor sections (yellow arrows point the distribution of SP-Au) at 7 h after SP-Au *i.v.* injection. (**b**) Frozen slices of tumor tissues stained with DAPI (blue, nuclei) and CD31 (green, vessels) at 7 h after SP-Au *i.v.* injection. The red signals were SP-Au. (**c**) Representative optical images and (**d**) fluorescence intensity of chlorophyll in urines collected at different time points (0, 3, 6, 12, 24, 48 and 72 h) after SP-Au *i.v.* injection

The intratumoral distribution of SP-Au was further analyzed using immunohistochemical staining (IHC) and immunofluorescence staining (IF). Most of SP-Au was confined within the CD31 + blood vessels, which was consistent with its micrometric size (Fig. 6a, b). A small portion of SP-Au extravasated and penetrated into the tumor parenchyma, but probably only after degradation. Indeed, both IHC and IF revealed the degradation of SP-Au into smaller fragments. To further validate this observation, we performed in vitro degradation by incubating SP with tumor cells for 6 h, which resulted in large quantities of smaller debris (Fig. S5) [54, 55]. We also collected urine from mice after SP-Au injection. Fluorescence signal was detected at 6 h post-injection (Fig. 6c, d). A chlorophyll-coloring of the urine was also observed within the same time frame. In summary, our results demonstrated that SP-Au could accumulate in tumor and be metabolized within 24 h of injection.

### In vivo radiosensitization of SP-Au

**Fig. 7:**
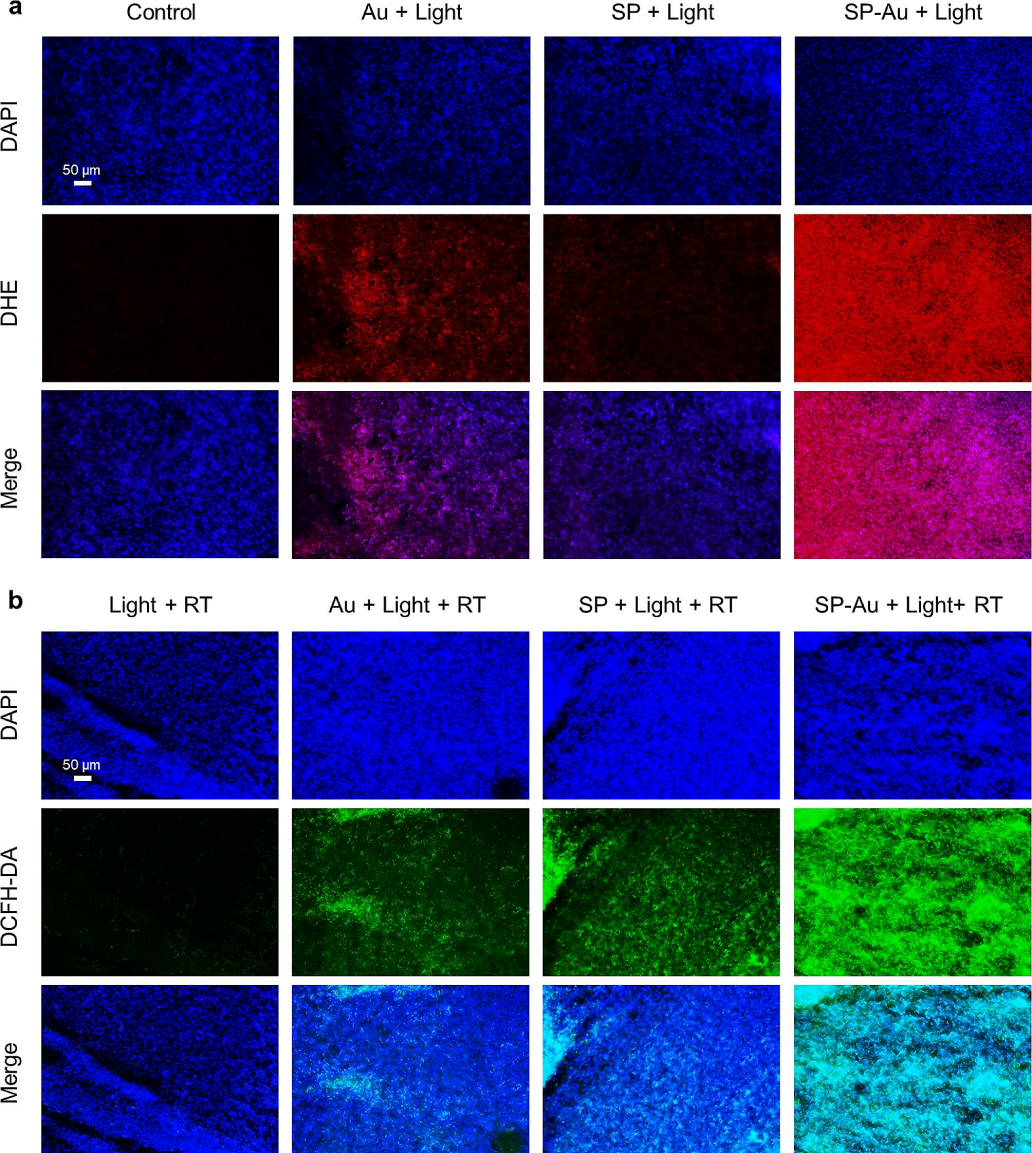
In vivo catalytic performance and radiosensitization efficiency of SP-Au. (**a**) Representative DHE-stained frozen slices of tumor tissues (red, •O_2_^−^) after *i.v.* injection of different catalyzers with red light treatment (615 ∼ 650 nm, 4600 lx, 15 min). (**b**) Frozen slices of tumor tissues stained with DAPI (blue, nuclei) and DCFH-DA (green, ROS) after *i.v.* injection of different catalyzers with red light illumination (615 ∼ 650 nm, 4600 lx, 15 min) and X-ray radiation (6 Gy)

The •O_2_^−^ and GSH levels in tumors were sampled and measured 30 min after Light or Light + RT treatment. Staining of tumor sections with DHE (red fluorescence, detecting •O_2_^−^) showed that the SP-Au + Light group had the highest amount of •O_2_^−^ (Fig. 7a), and the GSH content in the tumor was also reduced in the treatment group (Fig. S6). Accordingly, the DCFH-DA staining (green fluorescence, detecting ROS) revealed that the SP-Au + Light + RT group had the highest amount of ROS (Fig. 7b).

**Fig. 8:**
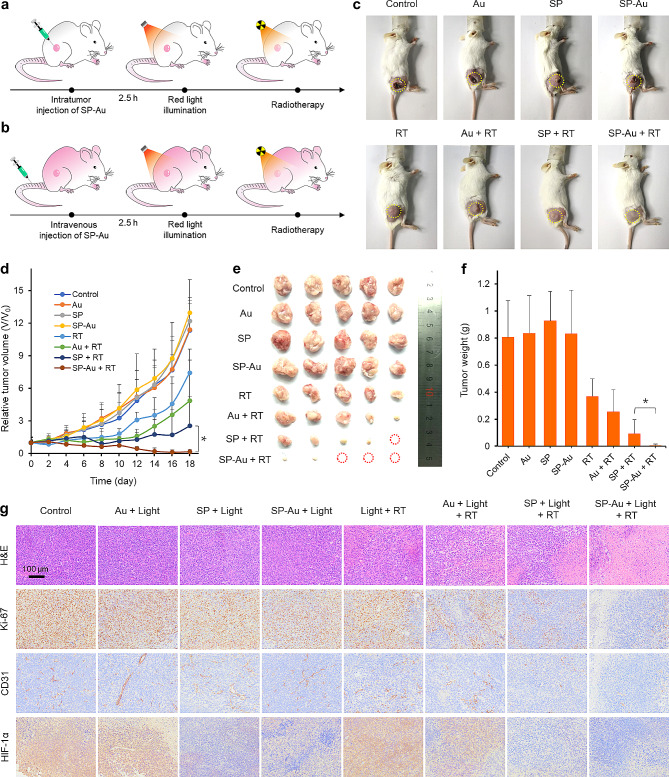
In vivo evaluation of intratumor SP-Au injection based multiway enhanced radiotherapy in 4T1 tumor-bearing mice. Schematic illustration for the multiway enhanced radiotherapy in (**a**) 4T1 or (**b**) A549 tumor-bearing mice. (**c**) Representative photographs of 4T1 tumor-bearing mice at day 18 after given various treatments. (**d**) Tumor growth curves of mice after given various treatments (*n = 5*). (**e**) Representative photograph of dissected tumors and (**f**) the tumor weights at day 18 after given various treatments (*n = 5*). (**g**) Representative H&E, CD31, Ki-67, and HIF-1*α* staining images of tumors. All data were presented as the mean ± standard deviation

The tumor growth curves in 4T1 models were recorded after treatments with different regimens. Au-SP, SP, or Au NCs were intratumorally injected (Fig. 8a). The anti-tumor efficacy of intravenously injected SP-Au was also studied in A549 models (Fig. 8b). While both Au and SP sensitized RT to some degree, the combination of SP-Au and RT effectively eliminated most of residual tumors at 18 days after the start of treatments, which corroborated with the results of tumor images, sizes, and weights (Fig. 8c-f). Pathological analysis and IHC staining revealed that tumor sections in the SP-Au + Light + RT group had the largest proportion of necrosis, with the lowest percentage of CD31 + blood vessels and Ki-67＋ cell (Fig. 8g and Fig. S7). Importantly, the expression of HIF-1*α* was lower in all groups containing SP and light treatment, underpinning its potent oxygenation capacity.

**Fig. 9:**
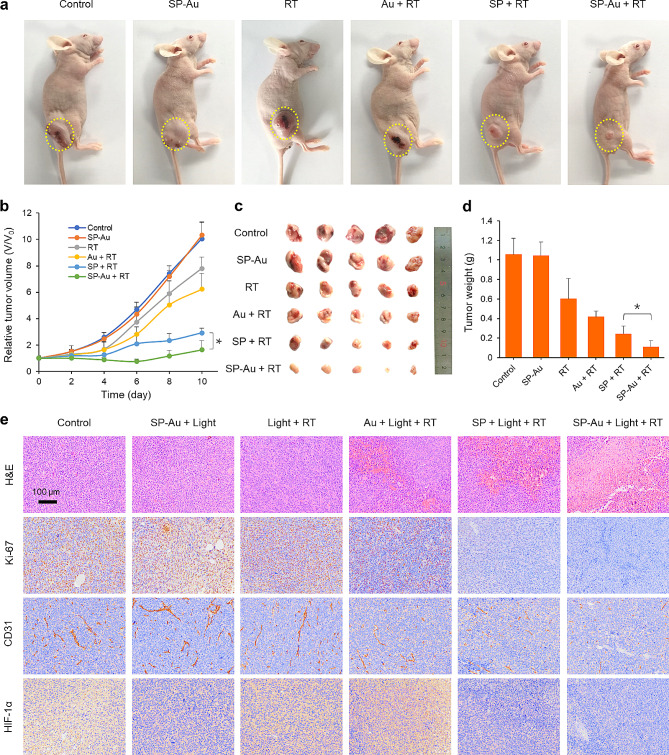
In vivo evaluation of intravenous SP-Au injection based multiway enhanced radiotherapy in A549 tumor-bearing mice. (**a**) Representative photographs of A549 tumor-bearing mice at day 10 after given various treatments. (**b**) Tumor growth curves of mice after given various treatments (*n = 5*). (**c**) Representative photograph of dissected tumors and (**d**) the tumor weights at day 10 after given various treatments (*n = 5*). (**e**) Representative H&E, CD31, Ki-67, and HIF-1*α* staining images of tumors. All data were presented as the mean ± standard deviation

Similar to the findings in Fig. 8, the combination of SP-Au and RT exhibited the best therapeutic efficacy in A549 tumor-bearing models by intravenous injection, further proving the radiosensitization capacity of SP-Au (Fig. 9a-d). Pathological analysis and the IHC staining of Ki-67, CD31, and HIF-1*α* also yielded similar trends with those of the 4T1 models (Fig. 9e and Fig. S8).

**Fig. 10:**
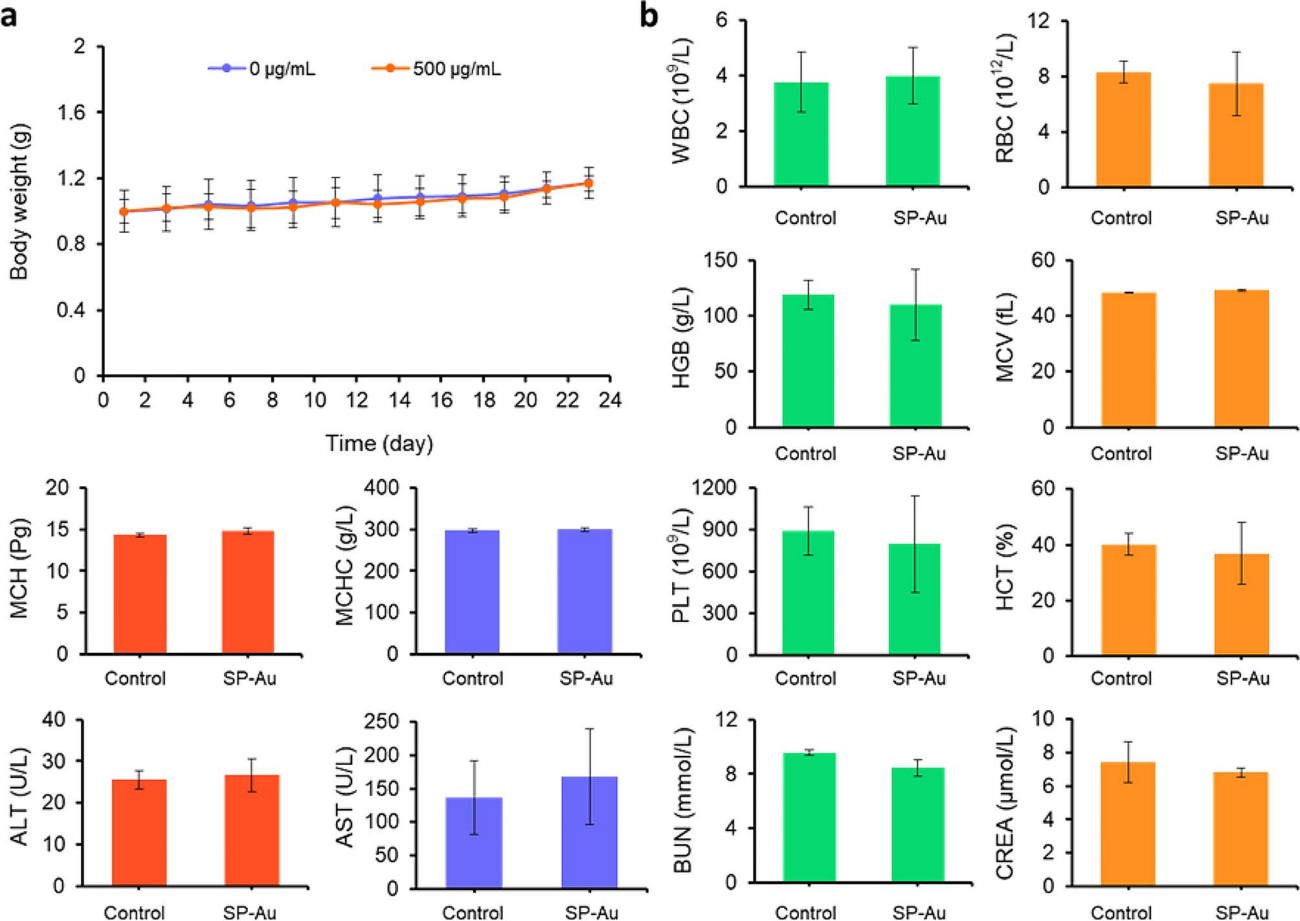
Preliminary toxicity analysis of SP-Au. (**a**) Body weight of mice (*n = 3*) after *i.v.* injection of SP-Au. (**b**) Blood routine and blood biochemistry tests of the mice (*n = 3*) after *i.v.* injection of SP-Au. All data were presented as the mean ± standard deviation

We next evaluated the in vivo toxicity of SP-Au. Intravenous injection of 500 µg/mL SP-Au, which was more than twice the therapeutic dose, did not cause any body weight loss (Fig. 10a), or any blood or biochemical indices (Fig. 10b). Pathological examination of major organs also revealed no significant alterations. These results proved that SP-Au can be used safely to sensitize radiation through multiple mechanisms (Fig. S9-S12).

## Conclusion

In summary, a new photosynthetic bacteria basis whole-cell inorganic-biohybrid system was developed to enhanced radiotherapy through multiple mechanisms. The resultant SP-Au accumulated in tumor after intravenous injection, followed by a rapid biodegradation and excretion through kidney. Under red-light irradiation, the SP-Au produced oxygen to ameliorate tumor hypoxia, which was then converted to •O^2−^ and further oxidized GSH. With the hypoxic regulation, •O^2−^ production, GSH oxidation, and the radiotherapy sensitization by gold nanoclusters, SP-Au substantially enhanced radiotherapy in both 4T1 and A549 tumor models with minimal toxicity. Therefore, the whole-cell inorganic-biohybrid system reported herein had substantial potential for the in clinical translation as a novel radiosensitizer.

### Electronic supplementary material

Below is the link to the electronic supplementary material.

Supplementary Material 1

## Data Availability

No datasets were generated or analysed during the current study.
