# Supplementary material for: Photosynthetic bacteria-based whole-cell inorganic-biohybrid system for multimodal enhanced tumor radiotherapy
Source: J Nanobiotechnology. 2024 Jun 28;22:379. doi: 10.1186/s12951-024-02654-7 (PMC11212166; doi:10.1186/s12951-024-02654-7)
Supplement: Supplementary file 1 — Supplementary Material 1 [file 12951_2024_2654_MOESM1_ESM.docx]

Photosynthetic bacteria-based whole-cell inorganic-biohybrid system for multimodal enhanced tumor radiotherapy

Shiyuan Hua ^a, e, f, 1^, Jun Zhao ^c,1^, Lin Li ^b,1^, Chaoyi Liu ^f^, Lihui Zhou ^h^, Kun Li ^d, *^, Quan Huang ^b, *^, Min Zhou ^a, e, f, g, *^, Kai Wang ^a, *^

^a^ Department of Respiratory and Critical Care Medicine, The Fourth Affiliated Hospital, Zhejiang University School of Medicine, Yiwu 322000, China

^b^ Department of Orthopedic Oncology, Spine Tumor Center, Changzheng Hospital, Naval Medical University, 415 Fengyang Road, Shanghai 200003, China.

^c^ School of Basic Medicine, Tongji Medical College, Huazhong University of Science and Technology, Wuhan, Hubei 430030, China.

^d^ Department of Musculoskeletal Oncology, Fudan University Shanghai Cancer Center, Shanghai, 200032, China

^e^ University-University of Edinburgh Institute (ZJU-UoE Institute), Zhejiang University School of Medicine, Zhejiang University, Haining, 314400, China.

^f^ Institute of Translational Medicine, Zhejiang University, Hangzhou, 310009, China.

^g^ Research Center for Life Science and Human Health, Binjiang Institute of Zhejiang University, Hangzhou, 310053, China.

^h^ Department of Neuro Surgery, The First Affiliated Hospital, Zhejiang University School of Medicine, Hangzhou 320000, China

^1^ These authors contributed equally to this work.

^*^ Corresponding author.

E-mail addresses: kaiw@zju.edu.cn (Kai Wang), zhoum@zju.edu.cn (Min Zhou), Kunli12345@163.com (Kun Li), huangquan0625@163.com (Quan Huang)

**Supplementary Fig.s**


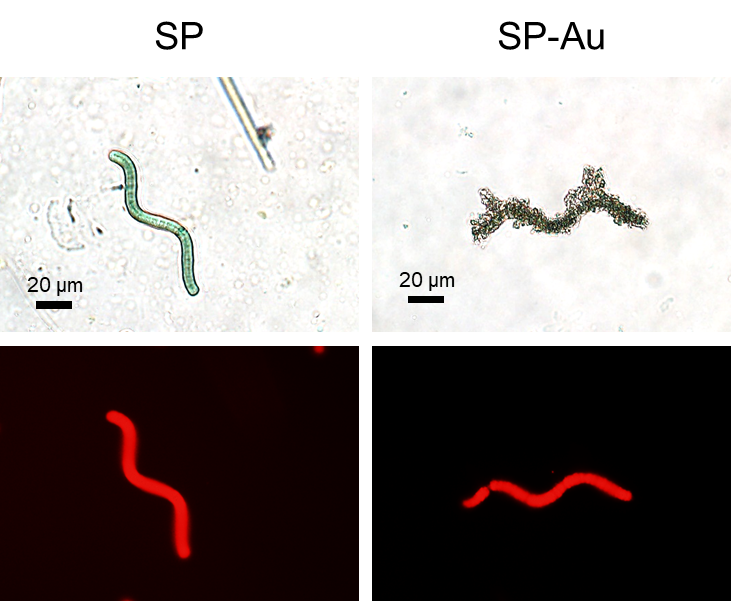


**Fig. S1.** Bright-field and fluorescence images of SP and SP-Au.


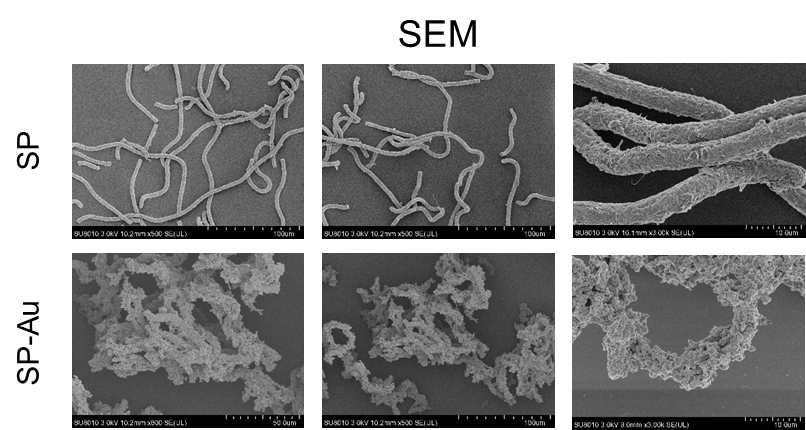


**Fig. S2.** SEM images of SP and SP-Au.


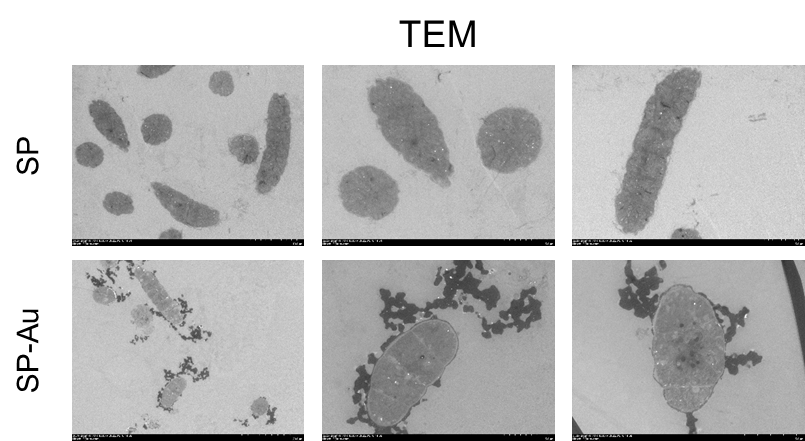


**Fig. S3.** TEM images of SP and SP-Au.


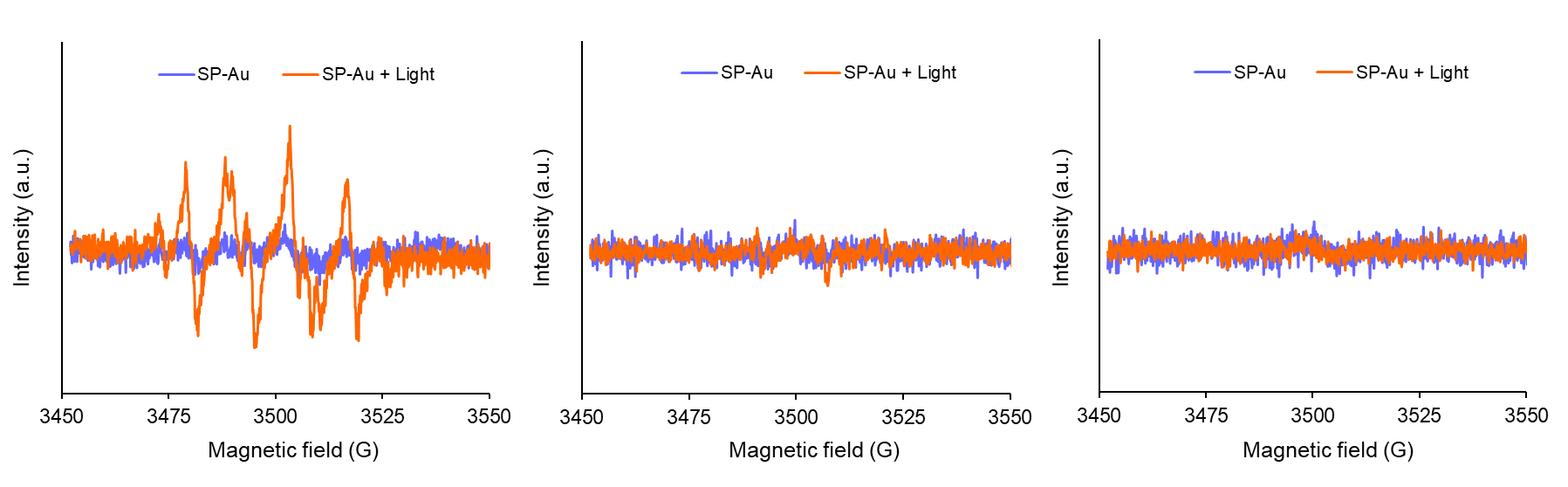


**Fig. S4.** ESR spectra of •O_2_^−^, •OH and ^1^O_2_ generated by SP-Au before and after red light illumination (4600 lux, 15 min).


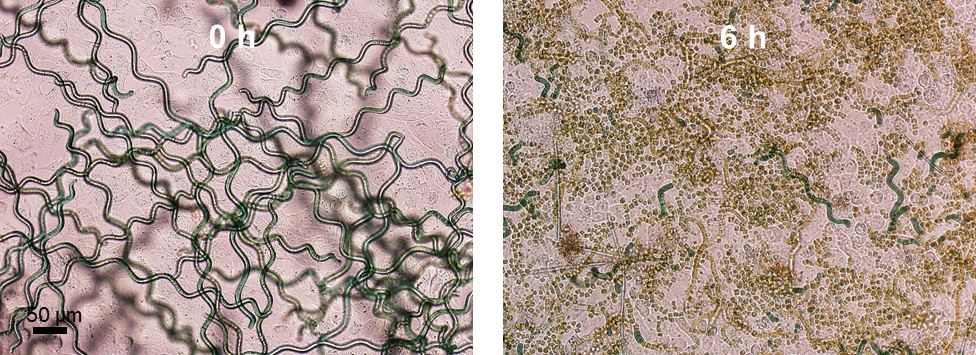


**Fig. S5.** Representative Bright-field images of SP before and after incubated with 4T1 cells for 6 h.


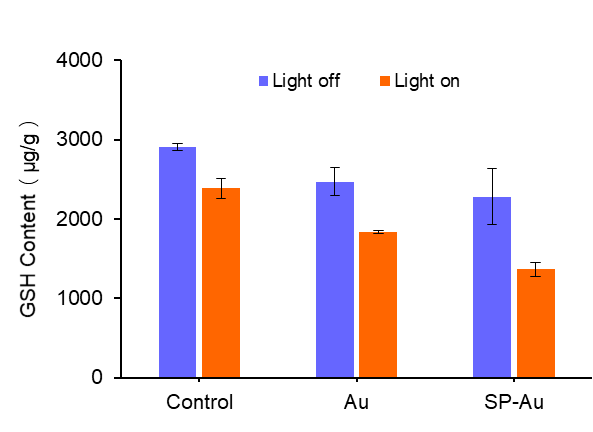


**Fig. S6.** GSH consumption in the tumor with different catalyzers before and after red light illumination (4600 lux, 15 min). All data were presented as the mean ± standard deviation.


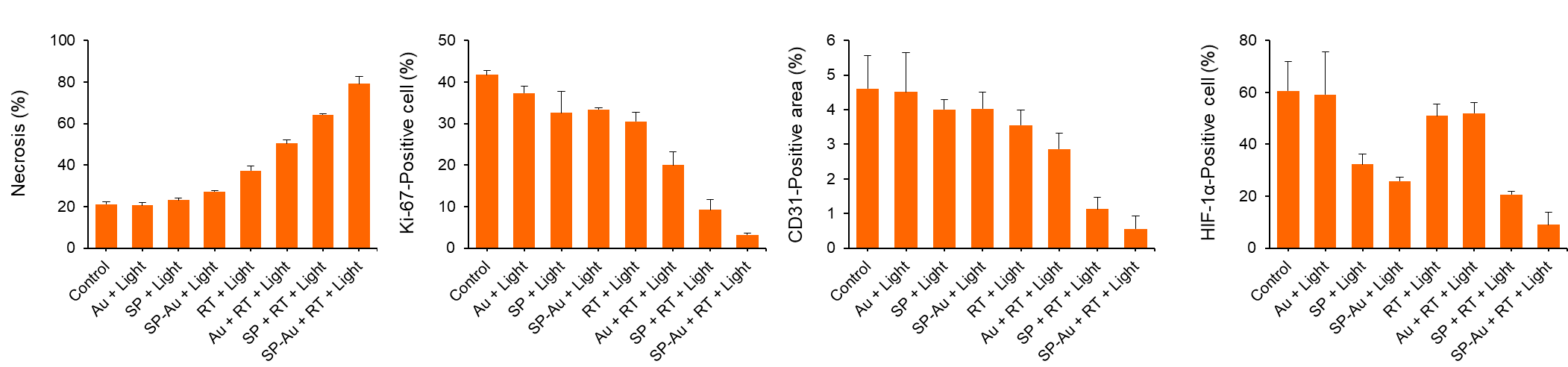


**Fig. S7.** The percentages of 4T1 tumor necrosis, Ki-67 positive cell, CD31 positive area and HIF-1*α* positive cell after different treatments. All data were presented as the mean ± standard deviation.


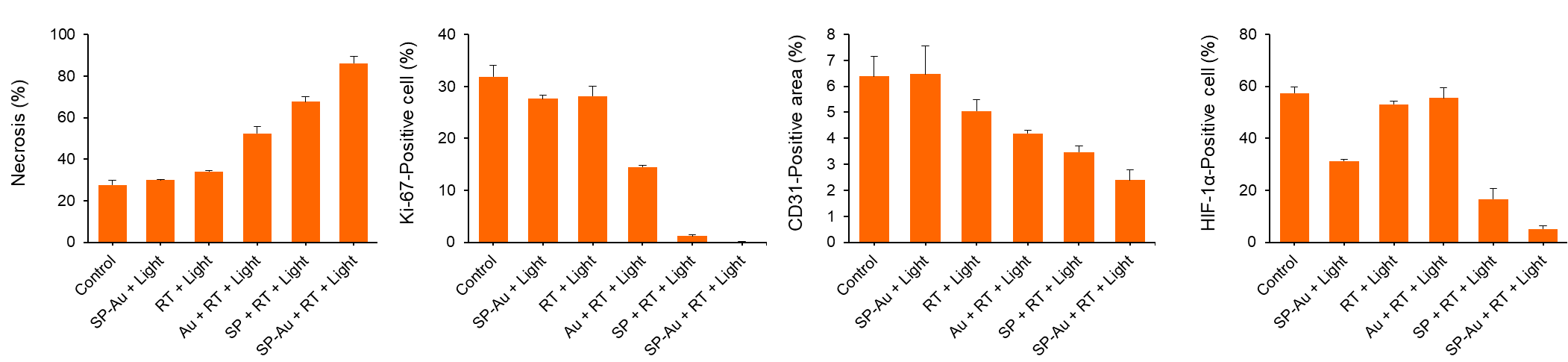


**Fig. S8.** The percentages of A549 tumor necrosis, Ki-67 positive cell, CD31 positive area and HIF-1*α* positive cell after different treatments. All data were presented as the mean ± standard deviation.


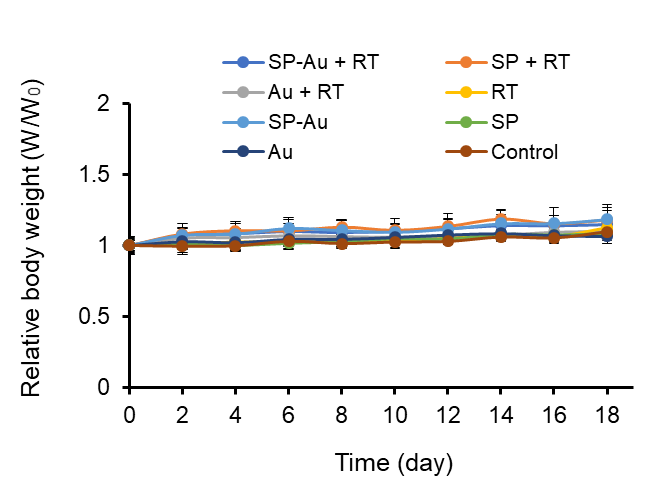


**Fig. S9.** Body weight of 4T1 tumor-bearing mice after given various treatments (*n = 5*). All data were presented as the mean ± standard deviation.


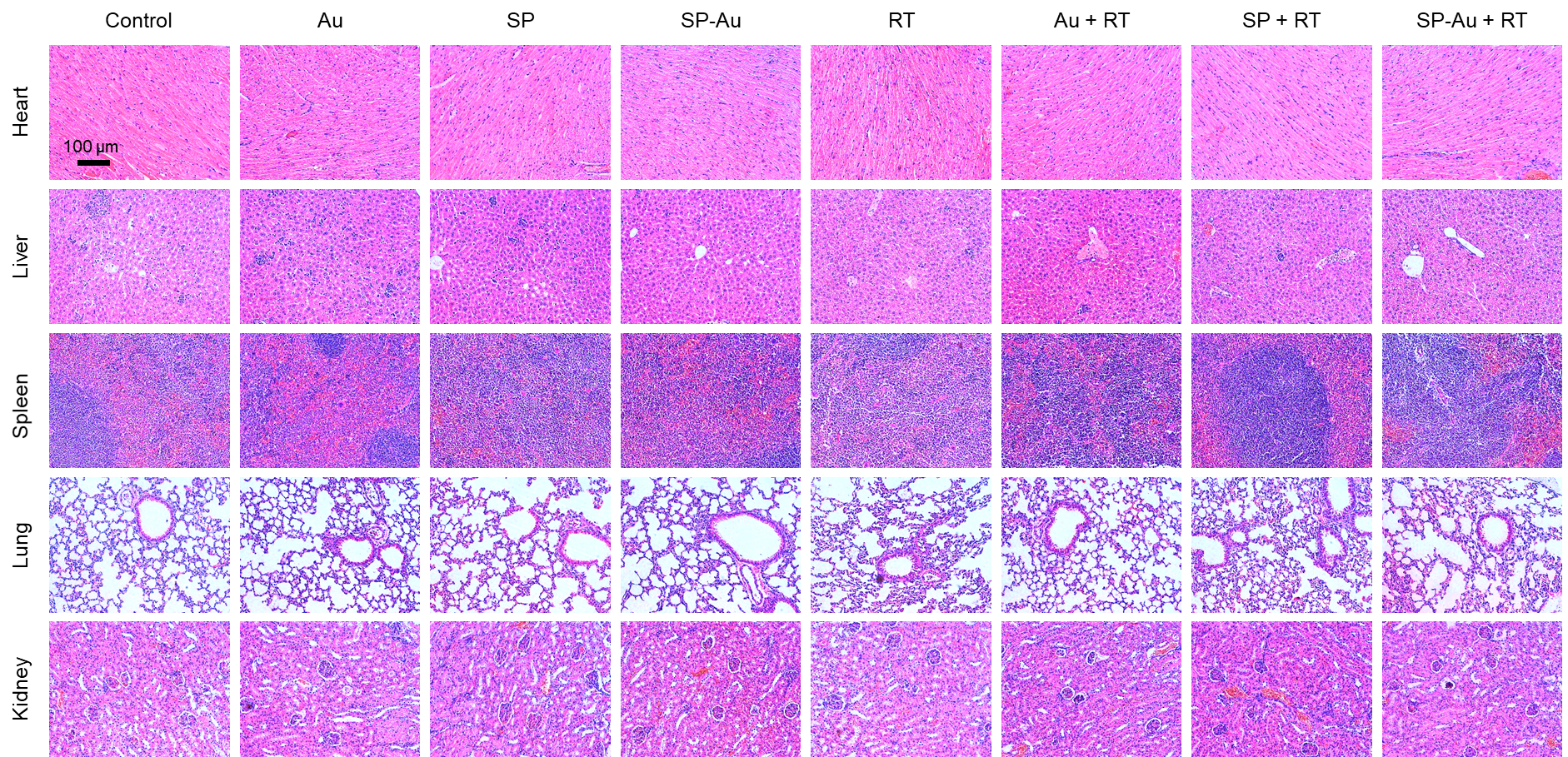


**Fig. S10.** Representative H&E staining images of major organs (heart, liver, spleen, lung, and kidney) from 4T1 tumor-bearing mice after given various treatments.


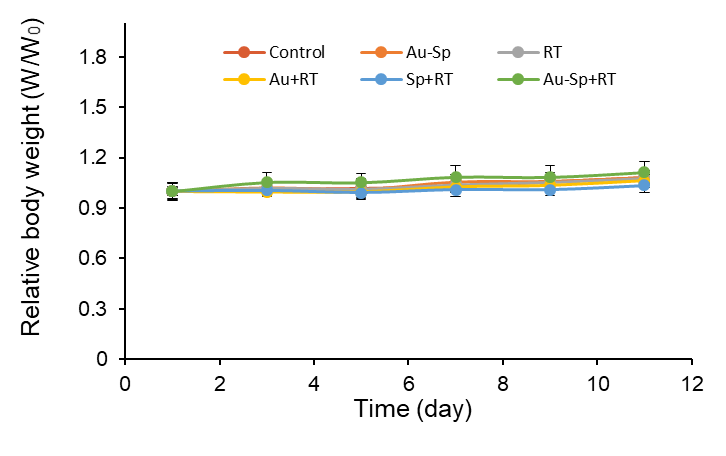


**Fig. S11.** Body weight of CT26 tumor-bearing mice after given various treatments (*n = 5*). All data were presented as the mean ± standard deviation.


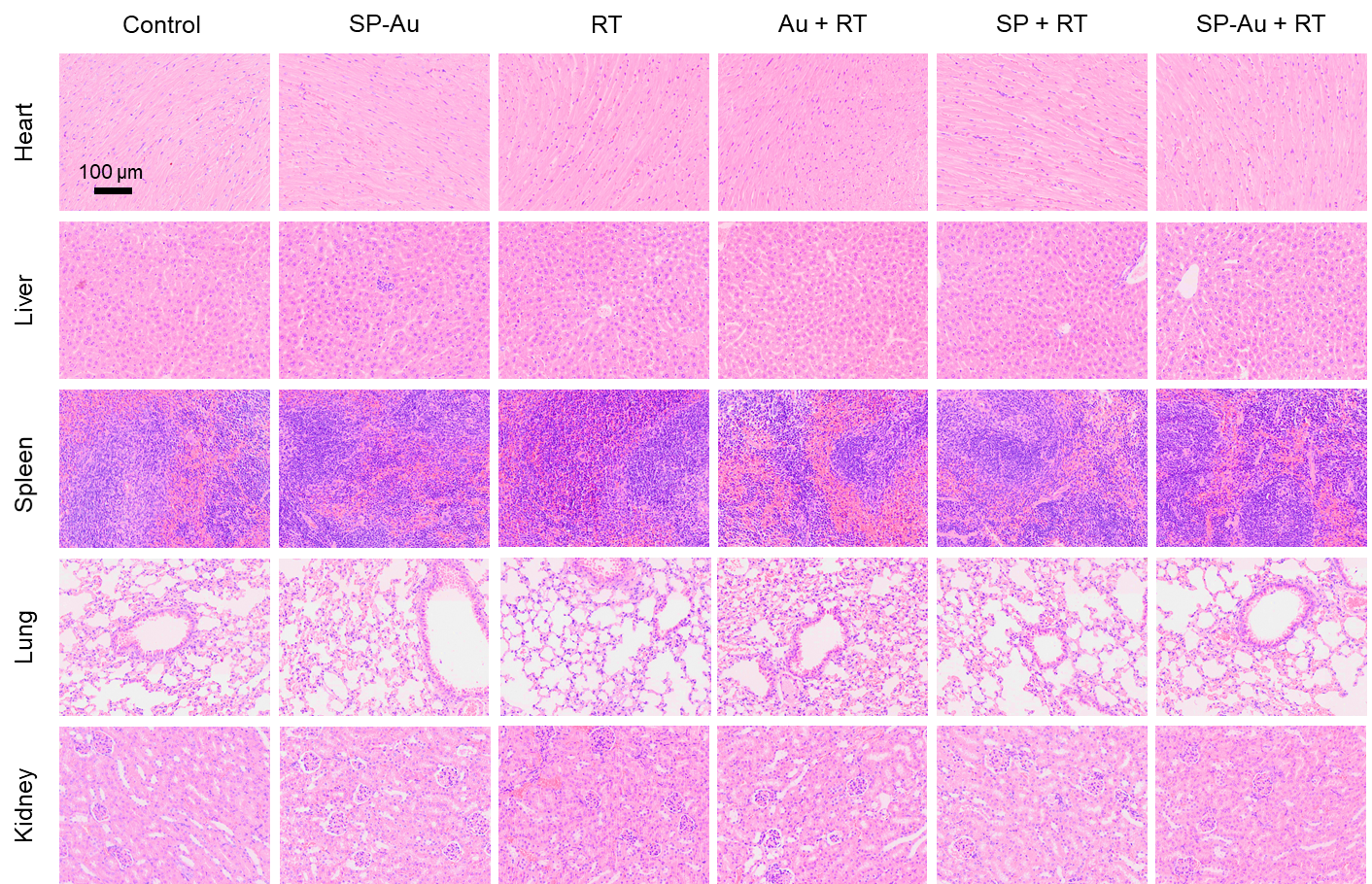


**Fig. S12.** Representative H&E staining images of major organs (heart, liver, spleen, lung, and kidney) from CT26 tumor-bearing mice after given various treatments.
